# Activity‐induced secretion of semaphorin 3A mediates learning

**DOI:** 10.1111/ejn.15210

**Published:** 2021-04-05

**Authors:** Aoi Jitsuki‐Takahashi, Susumu Jitsuki, Naoya Yamashita, Meiko Kawamura, Manabu Abe, Kenji Sakimura, Akane Sano, Fumio Nakamura, Yoshio Goshima, Takuya Takahashi

**Affiliations:** ^1^ Department of Physiology Yokohama City University Graduate School of Medicine Yokohama Japan; ^2^ Department of Molecular Pharmacology and Neurobiology Yokohama City University Graduate School of Medicine Yokohama Japan; ^3^ Department of Biochemistry Tokyo Women's Medical University School of Medicine Tokyo Japan; ^4^ Department of Pharmacology Juntendo University School of Medicine Tokyo Japan; ^5^ Department of Animal Model Development Brain Research Institute Niigata University Niigata Japan

**Keywords:** AMPA receptors, axonal guidance molecules, LTP, synaptic plasticity

## Abstract

The semaphorin family is a well‐characterized family of secreted or membrane‐bound proteins that are involved in activity‐independent neurodevelopmental processes, such as axon guidance, cell migration, and immune functions. Although semaphorins have recently been demonstrated to regulate activity‐dependent synaptic scaling, their roles in Hebbian synaptic plasticity as well as learning and memory remain poorly understood. Here, using a rodent model, we found that an inhibitory avoidance task, a hippocampus‐dependent contextual learning paradigm, increased secretion of semaphorin 3A in the hippocampus. Furthermore, the secreted semaphorin 3A in the hippocampus mediated contextual memory formation likely by driving AMPA receptors into hippocampal synapses via the neuropilin1–plexin A4–semaphorin receptor complex. This signaling process involves alteration of the phosphorylation status of collapsin response mediator protein 2, which has been characterized as a downstream molecule in semaphorin signaling. These findings implicate semaphorin family as a regulator of Hebbian synaptic plasticity and learning.

## Graphical abstract

Contextual fear learning task increased the secretion of semaphorin 3A, a secreted type of semaphorin, in the hippocampus. Furthermore, the secreted semaphorin 3A in the hippocampus mediated contextual memory formation by driving AMPA receptors into hippocampal synapses via the neuropilin 1/plexin A4 semaphorin receptor complex. Thus, the semaphorin family regulates both Hebbian synaptic plasticity and learning.

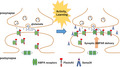

**Abbreviations**
ACSF: artificial cerebrospinal fluid
AMPA: α‐amino‐3‐hydroxy‐5‐methylisoxazole‐4‐propionic acid
ANOVA: analysis of variance
CRMP2: collapsin response mediator protein 2
DIV: day in vitro
ECL: enhanced chemiluminescence
EGFP: enhanced green fluorescent protein
EPSC: excitatory postsynaptic current
IA: inhibitory avoidance
LTP: long‐term potentiation
NMDA: *N*‐methyl‐d‐aspartate
NRP: neuropilin
P: postnatal day
PSD: postsynaptic density
Sema: semaphoring
SEP: super ecliptic pHluorin

## 1 INTRODUCTION

Semaphorins constitute one of the largest families of secreted and membrane‐bound molecules that are involved in the regulation of axonal pathfinding, cell migration, and immune systems (Kolodkin & Tessier‐Lavigne, 2011). Semaphorins are classified into several groups based on their structure (Jongbloets & Pasterkamp, 2014; Kolodkin & Tessier‐Lavigne, 2011). Semaphorin 3A (Sema3A) is a class 3 secreted‐type semaphorin and well characterized as a repulsive molecule of developing axons (Jongbloets & Pasterkamp, 2014; Luo et al., 1993). Neuropilin 1 (NRP1) provides the primary binding site for Sema3A and forms a complex with plexins, which possess a large intracellular domain and which transfer Sema3A signals (He & Tessier‐Lavigne, 1997; Kolodkin et al., 1997; Nakamura et al., 1998; Takahashi et al., 1998, 1999; Tamagnone et al., 1999). Although the physiological roles of semaphorins in activity‐independent axon guidance and neuronal cell migration have been intensively studied, the roles of semaphorins in activity‐dependent neuronal events, such as learning and memory, remain unclear.

Glutamatergic synapses play essential roles in activity‐dependent neuronal functions, such as learning and memory. Synaptic delivery of α‐amino‐3‐hydroxy‐5‐methylisoxazole‐4‐propionic acid (AMPA)‐type glutamate receptors is involved in Hebbian plasticity, which coincident presynaptic input and postsynaptic activity lead to changes in synaptic strength, and underlies many forms of learning and memory (Clem & Barth, 2006; Clem et al., 2008; Jitsuki et al., 2011, 2016; Kessels & Malinow, 2009; Lee et al., 2003; Mitsushima et al., 2011, 2013; Rumpel et al., 2005; Takahashi et al., 2003; Takemoto et al., 2017). AMPA receptors form a heteromeric tetramer composed of GluA1‐A4 (Bredt & Nicoll, 2003; Malinow & Malenka, 2002). We have previously shown that Sema3A regulates spine maturation and dendritic trafficking of AMPA receptors (Morita et al., 2006; Yamashita et al., 2014). PlexA4, a signal‐transducing component of Sema3A in vivo (Yaron et al., 2005), mediates retrograde Sema3A signaling from the axonal growth cone, acts as a cis‐interacting receptor with GluA2 in somatodendritic regions, and escorts GluA2 to the distal dendrites (Yamashita et al., 2014). Furthermore, we have previously reported that CRMP2 is a target molecule of edonerpic maleate, which facilitates synaptic AMPA receptor delivery and accelerates the effect of rehabilitation after brain damage (Abe et al., 2018). A recent study demonstrated that Sema3F/NRP2/PlexinA3 mediates downscaling of AMPA receptor‐mediated synaptic currents with a global increase in neuronal activity in vitro (Wang et al., 2017). Despite growing attention on semaphorins as regulators of neuronal plasticity, their roles in Hebbian plasticity and, most importantly, learning and memory, remain to be elucidated.

Here, we report that hippocampus‐dependent learning induced secretion of Sema3A in the rodent's hippocampus. Furthermore, in the rat model, learning‐driven secretion of Sema3A mediated synaptic AMPA receptor delivery at CA3–CA1 hippocampal synapses and contextual fear learning via the NRP1/plexin A4 complex, with decreased phosphorylation of CRMP2. While Sema3F/NRP2/PlexinA3 mediates synaptic scaling (Wang et al., 2017), Sema3A/NRP1/PlexinA4 is involved in Hebbian plasticity and mediates learning and memory. Thus, distinct Sema3/NRP/Plexin complexes play different roles in neuronal plasticity.

## 2 MATERIALS AND METHODS

### 2.1 Ethical statement

All animal experiments were conducted in strict accordance with the protocols approved by the Institutional Animal Care and Use Committee of Yokohama City University (authorization number: F‐A‐18‐009, F‐A‐20‐011). All surgical procedures were performed under anesthesia, and every effort was made to minimize the sufferings.

### 2.2 Animals

Male Sprague–Dawley (SD) rats (postnatal 4–5 weeks of age), male C57BL/6J mice (postnatal 7–11 weeks of age), and Flag‐SEP‐Sema3A knock‐in mice (postnatal 7–11 weeks of age) were used (Niigata University: Comprehensive Brain Science Network). Animals were housed on a constant 14‐hr light/10‐hr dark cycle in plastic cages. Food and water were provided for ad libitum consumption. Procedures were performed in strict compliance with the animal use and care guidelines of Yokohama City University.

### 2.3 Cell culture

Hippocampal neurons from male and female ICR E16 (Charles River Laboratories, Japan) or male and female Wistar rat E17‐18 (Charles River Laboratories) were cultured on 0.01% poly‐L‐ornithine (Wako Pure Chemicals, Japan)‐coated dishes in neurobasal medium (Gibco, U.S.A) supplemented with 2% B27 supplement (Gibco, U.S.A) and 0.5mM L‐glutamine (Nacalai Tesque, Japan). Neurons were cultured for 21–28 days in vitro (DIV) on plastic coverslips.

### 2.4 Constructs

The plexin A4 and its mutant constructs were prepared as previously described (Yamashita et al., 2014). For knockdown of plexin A4, NRP1, and NRP2, short hairpin (Sh) RNAs were subcloned into the pFUGW vector. The target sequences for the Sh RNAs were as follows: Plexin A4 (Yamashita et al., 2014): 5′‐GAGCAAGCTAGAGTATGCCACTGAT‐3′, NRP1: 5′‐TATAGTTCTGAGAACATTCGG‐3′, NRP2 (Chen et al., 2008): 5′‐GCTATGACATGGAGTATCA‐3′, shPlexA4 scramble: 5′‐TAGTCACCGTATGAGATCGAACGAG‐3′, Negative ctrl shRNA: 5′‐GGTAAGTGCCCAAATATCT‐3′.

### 2.5 Immunocytochemistry of cultured neurons

After treatment with recombinant Sema3A (0.1 nM) (Yamashita et al., 2014) for 30 min, cultured neurons were fixed with 4% paraformaldehyde (PFA). To visualize the surface level of GluA1, immunocytochemistry without TritonX‐100 permeabilization was performed. To visualize the total level of GluA1, plexin A4, MAP2, and GFP, immunocytochemistry was performed using a permeabilization protocol with 0.1% TritonX‐100. For staining the cultured neurons, the antibodies used were as follows: anti‐N‐terminal GluA1 mouse monoclonal antibody (1:500 dilution; EMD Millipore, USA), anti‐MAP2 rabbit polyclonal antibody (PRB‐547C, 1:1,000 dilution; Covance, USA), anti‐MAP2 mouse monoclonal antibody (M1406, 1:1,000 dilution; Sigma, USA), anti‐GFP chicken antibody (1:1,000 dilution; Aves), anti‐NRP1 hamstar (1:500 dilution, provided by Dr. F. Suto), anti‐NRP2 hamstar (1:500 dilution, provided by Dr. F. Suto), and anti‐PlexA4 hamster monoclonal antibody (1:500 dilution, provided by Dr. F. Suto; Suto et al., 2007). The neurons were counter‐stained with anti‐MAP2 rabbit polyclonal antibody. We used a laser scanning microscope (Olympus FV‐1000). Immunostained neurons were captured with a 40× objective (numerical aperture 0.95), while high‐magnification images with a 60× oil immersion lens (numerical aperture 1.42), with imaging software (FV10‐ASW). For measuring the mean intensities after immunostaining with anti‐GluA1 and anti‐plexin A4 antibodies, ImageJ software was used. To obtain the mean intensity of dendrite per neuron, the intensity of each MAP2‐ or GFP‐positive dendrites was normalized to the volume of dendrites. The relative immunostaining intensity of each area was calculated.

### 2.6 Immunoprecipitation

HEK293T cells were seeded at 4.0 × 10^5^ cells/dish in a 6‐cm dish. One day later, the cells were transfected with EGFP‐GluA1, together with full‐length plexin A4, Δect, IPT domain (Yamashita et al., 2014), and the plexinA4‐Myc using transfection reagent (FuGENE6, Promega). After 48 hr of transfection, cells were lysed by immunoprecipitation buffer (20 mM Tris‐HCl [pH 8.0], 100 mM NaCl, 10 mM NaF, 1 mM Na_3_VO_4_, 1 mM EDTA, 1% Nonidet P‐40, and cOmplete Mini protease inhibitor cocktail [Roche]), and then immunoprecipitated with 2 µg of anti‐Myc mouse monoclonal antibody (M5546; Sigma, USA).

In experiment using animal brain, postnatal day (P) 5, P10, and P15 rat hippocampal samples were homogenized in immunoprecipitation buffer. The lysates were centrifuged at 20,000 *g* for 20 min at 4°C. The supernatants were then incubated with 1 µg of anti‐plexin A4 mouse monoclonal antibody (Suto et al., 2007) or control IgG, overnight at 4°C, followed by additional incubation with protein G magnetic beads (GE Healthcare, USA) for 1 hr at 4°C. After washing three times with immunoprecipitation buffer, samples were boiled in SDS buffer for 5 min at 100°C.

In experiment using primary cultured neurons, DIV21 neurons were treated with recombinant Sema3A (0.1 nM) for 30 min. After Sema3A stimulation, samples were homogenized in immunoprecipitation buffer. The lysates were centrifuged at 20,000 *g* for 20 min at 4°C. The supernatants were then incubated with 1 µg of plexin A4 antibody–Dynabeads (Thermo Fisher Scientific, USA) complex for 1 hr at room temperature. After washing with PBS, samples were boiled in SDS buffer for 5 min at 100°C.

### 2.7 Preparation of postsynaptic density fractions

Postsynaptic density (PSD) fractions were prepared as described previously (Tada et al., 2016). Dounce homogenate was prepared from the hippocampus and centrifuged at 1,000 *g* for 10 min to remove nuclei and debris (P1). The supernatant was spun at 12,000 *g* for 20 min to obtain a P2 fraction. P1 and P2 fractions were resuspended and centrifuged twice to remove contaminants. The P2 fraction was then resuspended in buffer containing 0.5% Triton X‐100 and rotated for 15 min. This fraction was then centrifuged at 100,000 *g* for 60 min to yield soluble and insoluble fractions (PSD fraction), and the insoluble fraction was then solubilized into T‐PER (Thermo Fisher Scientific, USA). All fractionation steps were performed at 4°C in the presence of 0.32 M sucrose and 4 mM Hepes, containing phosphatase inhibitor (Nacalai tesque, Japan) and complete protease inhibitor mixture (Roche, Swiss).

### 2.8 Western blotting

Samples were separated using 4%–15% gradient gel (Biorad, USA) and proteins were transferred to PVDF membranes. Membranes were blocked with 1% blocking buffer (Perfect‐block; MoBiTec, Germany) in 0.1% TBS‐T for 1 hr and incubated overnight at 4°C with primary antibodies: anti‐c‐Myc (1:5,000 dilution; Santa Cruz Biotechnology, USA), anti‐GFP (1:5,000; Santa Cruz Biotechnology), anti‐GluA1 (1:1,000; EMD Millipore), anti‐PlexA4 (1:1,000; Abcam), anti‐Phosphorylated Ser522 of CRMP1/2 (1:1,000; Wako), and anti‐β‐actin (1:5,000; Sigma). Membranes were subsequently washed in TBS‐T and placed in HRP‐conjugated anti‐rabbit secondary antibody at a 1:1,000 dilution. After washing, membranes were reacted with ECL or ECL‐prime reagents (GE Healthcare, USA). The chemiluminescence signals on the ECL‐treated membrane were detected using LAS4000 (Fujifilm, Japan).

### 2.9 Immunohistochemistry of brain slices

Mice (SEP Sema3A^KI/KI^ mice) were deeply anesthetized and perfused transcardially with phosphate‐buffered saline (PBS), followed by 4% paraformaldehyde (PFA) after 10 min of IA conditioning. Brains were extracted and incubated in 4% PFA at room temperature for 2 hr. Brain samples were transferred to PBS and 50‐μm coronal slices were prepared using a vibratome (Leica VT‐1000). For immunostaining of secreted Sema3A or surface GluA1, slices were stained without TritonX‐100 permeabilization. Slices were placed in PBS, with 3% normal goat serum for 2 hr and then incubated with primary antibody at 4°C for 24 hr. Slices then underwent three washing steps for 10 min each in PBS, followed by a 1‐hr incubation with secondary antibody. Slices were washed three more steps of 10 min each in PBS after incubation of secondary antibody. After staining of secreted Sema3A or surface GluA1, the slices were placed in PBS +0.2% Triton X‐100 (PBS‐T), with 3% normal goat serum for 2 hr, and then incubated with primary antibody at 4°C for 24 hr. Slices then underwent three washing steps for 10 min each in PBS‐T, followed by a 1‐hr incubation with secondary antibody. After three more washing steps of 10 min each in PBS‐T, slices were mounted on microscope slides. Antibodies used for staining were as follows: anti‐N‐terminal GluA1 mouse monoclonal antibody (1:500 dilution; EMD Millipore, USA), anti‐synapsin1 (1:1,000 dilution; Millipore), and anti‐GFP chicken antibody (1:1,000 dilution; Aves). We used a laser scanning microscope (Olympus FV‐1000, Japan). Brain slices were captured with a 40× objective (numerical aperture 0.95), while high‐magnification images of the hippocampal basal dendrites with a 100× oil immersion lens (numerical aperture 1.40), with imaging software (FV10‐ASW). For measuring the number of puncta after immunostaining with anti‐GFP, anti‐GluA1, and anti‐synapsin1 antibodies, Fluoview software (Olympus) was used.

### 2.10 Generation of FLAG‐SEP Sema3A^KI/KI^ mice

For generation of the flag‐SEP knock‐in mice, we designed a targeting construct in which flag‐tagged super ecliptic pHluorin cDNA (flag‐SEP) was placed just behind a secretion signal coding sequence (25 aa) of the Sema3A. The Quick and Easy BAC modification Kit (Gene Bridges, Dresden, Germany) was used for targeting vector construction. In brief, we retrieved a 11.92 kb homology arm from a genomic clone (RP23‐181I7) of C57/BL6 BAC library (advance GenoTechs, Tsukuba, Japan) and subcloned it into pDT‐MC#3 containing a CAG promotor‐driven diphtheria toxin gene. Then, we inserted a knock‐in fragment [flag‐SEP, sequence encoding 12 amino acids of exon2 followed by 608 bp of intron2‐3, and FRT‐flanked neomycin‐resistance cassette (Neo)] at the precise position of the homology arm of the pDT‐MC#3. Culture of ES cells, identification of recombinant ES cells, and generation of chimeric mice were performed as described previously (Mishina & Sakimura, 2007). After crossing with a FLP‐expressing mouse to eliminate the Neo cassette from the *Sema3A* locus, we established Sema3A^flag‐SEP^ mice. PCR genotyping of mouse tail DNA was performed with the following primers: Sema3AF, 5′‐AAGTTTCTGTGATTCCGTGACTGT‐3′; Sema3AR, 5′‐AGCAAGCACACAGCTAGCTCACTG‐3′; and SEP R, 5′‐CTTGAATTCTTTGTATAGTTCATC‐3′.

### 2.11 In vivo infection of hippocampal CA1 region

Rats were anesthetized with a mixture of isoflurane–oxygen. The skin overlying the skull was incised and gently pushed to the side. The region above the target area was gently pierced with a dental drill. The injection coordinates for the CA1 hippocampus were 3 mm posterior to bregma, 2 mm lateral to midline, and 2.5 mm depth from the brain surface. Recombinant lentivirus was injected using a pulled‐glass capillary (Duramond) into the CA1 hippocampus at a titer of 8 × 10^9^ TU/ml and a volume of 0.3 µl. Single injection into the right hippocampus was performed for whole‐cell recordings. For behavioral analyses, the viral solution was bilaterally injected.

### 2.12 Injection of anti‐Sema3A antibody

Rats were anesthetized with a mixture of isoflurane–oxygen. A guide cannula (AG‐4; EICOM, Kyoto, Japan) was implanted into CA1 region of the hippocampus. The implantation coordinates were 3.5 mm posterior to bregma, 2.5 mm lateral to midline, and 2.15 mm depth from the brain surface. Seven days after surgery, injection of anti‐Sema3A antibody solution 4‐2 hIgG and control IgG (Yamashita et al., 2015) was delivered via a Teflon tube through the guide cannula by application of pressure.

### 2.13 Inhibitory avoidance training

Animals were placed in a shielded room containing an inhibitory avoidance (IA) training apparatus on the training day. The apparatus was a two‐chambered box consisting of a lighted box (safe side) and a dark box (shock side), divided by a trap door (O'Hara, Japan). During training, animals were placed in the lighted side, away from dark side. After opening the trap door, the animal entered into the dark side at will. The latency for entering the dark side was determined as a behavioral parameter. Four seconds after the animals entered the dark side, the door was closed and applied scrambled electrical foot shocks (0.35 mA for 2 s: mice, 1.6 mA for 2 s: rats) via electrified steel rods situated in the floor of the box. Animals were left in the dark compartment for 10 s before being returned to their home cages. Thirty minutes after the learning experience, animals were placed in the light side of the box again. The latency to enter the experienced dark side was determined as learning performance.

### 2.14 Electrophysiology

Five to seven days after lentivirus injection, animals were anesthetized with isoflurane. The Brains were immediately transferred into bubbled (95% O_2_ and 5% CO_2_) ice‐cold dissection buffer as described previously (Abe et al., 2018; Jitsuki et al., 2016; Tada et al., 2015). Coronal brain slices were cut with a vibratome (350 μm; Leica VT1200) in dissection buffer. After cutting, the slices were then incubated in artificial cerebrospinal fluid (ACSF) as described previously (Abe et al., 2018; Jitsuki et al., 2016; Miyazaki et al., 2020). The recording chamber was perfused with an ACSF containing 0.1 mM picrotoxin and 4 mM 2‐chloroadenosine at 22°C–25°C. Patch pipettes (4–8 MΩ) for whole‐cell recordings were filled with intracellular solution (Jitsuki et al., 2016; Takahashi et al., 2003). Recordings were obtained from infected CA1 pyramidal neurons of the rat hippocampus with a patch‐clamp amplifier (MultiClamp 700B; Molecular Devices, USA). Stimulating electrodes were placed at the Schaffer collateral at least 200 μm away from recording neurons. Stimulus intensity was increased until a synaptic response of amplitude >ca. 10 pA was recorded. AMPA/NMDA ratios were calculated as the ratio of the peak current at −60 mV to the current of 50 ms after stimulus onset at +40 mV (30–50 traces averaged for each holding potential).

In experiments analyzing LTP, lentivirus‐infected slices were maintained in ACSF as described above. EPSC was evoked at 0.33 Hz (before pairing) or 0.1 Hz (after pairing) and recorded at −60 mV holding potential. LTP was induced by a standard pairing protocol (Chen et al., 1999; Jitsuki et al., 2016; Tada et al., 2015) consisting of 5‐Hz presynaptic stimulation with postsynaptic depolarization at 0 mV for 90 s. Recordings were maintained for at least 30 min after LTP induction. The EPSC amplitudes throughout the recording were normalized to the average amplitude before LTP induction.

### 2.15 Statistical analysis

To choose the appropriate statistical tests, the skewness and kurtosis of sample distribution were calculated. If the skewness was less than 2 and kurtosis was less than 7, we analyzed the data using parametric tests. When the data showed that either skewness was more than 2 or kurtosis was more than 7, we used non‐parametric tests (Kim, 2013; West et al., 1995). As parametric tests, an unpaired two‐tailed *t*‐test was used to compare two independent groups and one‐way ANOVA with post‐hoc Dunnett's test was used to compare more than three groups. For analyzing the effect of two factors, two‐way ANOVA with post‐hoc Sidak's multiple comparisons test was used. As non‐parametric tests, a Mann–Whitney U‐test was used to compare two independent groups. *p* < 0.05 was considered statistically significant. Statistical analyses were conducted using GraphPad Prism 7 (Graph Pad Software). In the box plot graphs, the ends of the whiskers were defined by maximum and minimum values. Central rectangles spanned from the first quartile to the third quartile. The segment in the rectangle indicated the median. In graphs other than box plot graphs, the error bars indicated standard error of the mean.

## 3 RESULTS

### 3.1 Sema3A regulates trafficking of AMPA receptors to the neuronal surface of hippocampal primary cultures

**FIGURE 1:**
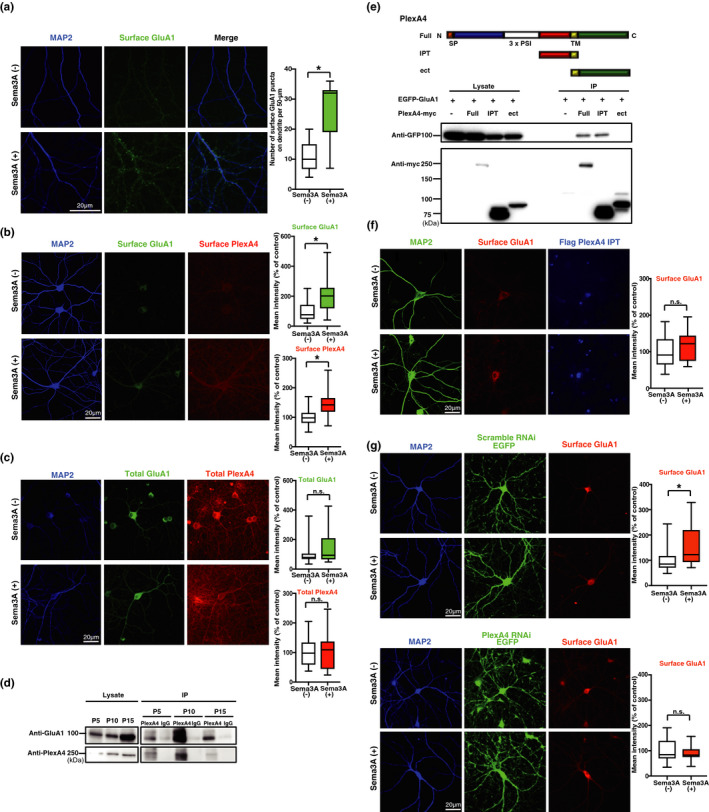
Sema3A regulates trafficking of AMPA receptors to the neuronal surface of hippocampal primary cultures. (a) (Left) Immunocytochemical images of surface GluA1 puncta in a MAP2‐positive dendrite of cultured hippocampal neurons treated with Sema3A(+) or control(‐). (Right) Quantitative analysis of surface GluA1 puncta on a dendrite. Data were obtained from 19 neurons (Sema3A) or 18 neurons (control) from three independent cultures. (b) (Left) Immunocytochemical images of surface plexin A4 and surface GluA1 in MAP2‐positive dendrites of cultured hippocampal neurons treated with Sema3A(+) or control(‐). (Right) Quantitative analysis of mean immunostaining intensity of surface plexin A4 and surface GluA1 on dendrites. Data were obtained from 20 neurons (Sema3A) or 20 neurons (control) from three independent cultures. (c) (Left) Immunocytochemical images of total plexin A4 and total GluA1 in the MAP2‐positive dendrite of cultured hippocampal neurons treated with Sema3A(+) or control(‐). (Right) Quantitative analysis of mean immunostaining intensity of surface plexin A4 and surface GluA1 on dendrites. Data were obtained from 25 neurons (Sema3A) or 26 neurons (control). (d) Immunoprecipitation of GluA1 and plexin A4 at postnatal day (P) 5, P10, or P15 rat hippocampal lysates. GluA1 and plexin A4 were co‐immunoprecipitated from rat hippocampal lysates at all examined ages. (e) (Top) Structure of plexin A4 and schematic representation of constructed plexin A4 mutant. Plexin A4 contains signal peptides, a sema domain, three PSI domains (domains found in plexin, semaphorin, and integrin), three immunoglobulin‐like plexin transcription factor (IPT) domains, transmembrane domain, and cytosolic domain. Abbreviations are as follows: Full, full length; IPT, three IPT; Δect, lacking the entire ectodomain. (Bottom) Immunoprecipitation of EGFP‐GluA1 and full‐length, Δect or IPT domain of plexin A4. EGFP‐tagged GluA1 interacted with the IPT mutant of plexin A4‐Myc. (f) (Left) Immunocytochemical images of surface GluA1 puncta in the MAP2‐positive dendrite of the plexin A4‐IPT domain‐expressing neurons treated with Sema3A(+) or control(‐). (Right) Quantitative analysis of surface GluA1 puncta on dendrite of plexin A4‐IPT domain‐expressing neurons. Data were obtained from 15 neurons (Sema3A) or 15 neurons (control) from three independent cultures. (g) (Left) Immunocytochemical images of surface GluA1 puncta in the MAP2‐positive dendrite of plexin A4 RNAi‐ or scramble RNAi‐expressing neurons treated with Sema3A(+) or control(‐). (Right) Quantitative analysis of surface GluA1 puncta on dendrites of plexin A4 RNAi‐ or scramble RNAi‐expressing neurons. Data were obtained from 22 to 25 neurons (Sema3A) or 24 to 26 neurons (control) from four independent cultures. *p < 0.05

We have previously shown that Sema3A regulates dendritic localization of GluA2/GluA3 heteromers in the early developmental stage (Yamashita et al., 2014). This raised the question as to whether Sema3A controls synaptic functions. To investigate whether Sema3A modifies synaptic plasticity, we first analyzed primary cultures of hippocampal neurons (day in vitro [DIV] 21–27) using immunocytochemical methods. We focused on the GluA1 subunit, as plasticity‐inducing stimuli, both in vitro and in vivo, primarily drive GluA1 into synapses (Hayashi et al., 2000; Shi et al., 2001; Takahashi et al., 2003). Immunostaining with an anti‐GluA1 antibody (N‐terminal) without permeabilization revealed that addition of purified Sema3A (see methods for details) increased the number of dendritic‐surface GluA1 puncta in primary cultures of hippocampal neurons (*p* < 0.0001, *t* = 6.566, *df* = 35) (Figure 1a). Interestingly, Sema3A stimulation increased surface plexin A4 (*p* = 0.0009, *t* = 3.587, *df* = 38), while total protein levels of GluA1 and plexin A4 remained unchanged after the addition of Sema3A (GluA1 *p* = 0.289, Mann–Whitney *U* = 263; plexin A4 *p* = 0.7201, *t* = 0.3604, *df* = 49) (Figure 1b,c). To investigate the relationship between Sema3A signaling and GluA1 cell‐surface trafficking further, we performed immunoprecipitation of hippocampal brain lysates at postnatal (P) days 5, 10, 15, and 28. We found that GluA1 co‐immunoprecipitated with plexin A4 at all examined ages (Figure 1d, Figure S1a), as was previously reported for GluA2 (Yamashita et al., 2014), indicating that GluA1 forms a complex with plexin A4.

We also found that Sema3A stimulation showed no effect on the formation of GluA1 and plexin A4 complex (*p* = 0.7762, *t* = 0.2974, *df* = 6) (Figure S1b). We previously reported that GluA2 and plexin A4 bound to each other through the immunoglobulin‐like plexins transcription factors (IPT) domain of plexin A4 (Yamashita et al., 2014). We thus tested whether GluA1 and plexin A4 also form a complex through the IPT domain of plexin A4. We overexpressed green‐fluorescent protein (GFP)‐tagged GluA1 with myc‐tagged full‐length plexin A4 or the myc‐tagged IPT domain of plexin A4 by lipofection‐mediated transfection of HEK293 cells (Figure 1e). We prepared cell lysates from these cells and performed immunoprecipitation using an anti‐myc antibody, and immunoblotting with an anti‐GFP antibody. We found that the plexin A4 IPT domain bound to GluA1 (Figure 1e). We next examined whether interaction between GluA1 and plexin A4 via the IPT domain is responsible for the Sema3A‐induced increased trafficking of GluA1 to the neuronal surface. We overexpressed the IPT domain of plexin A4 by electroporation and examined the effect of Sema3A on cell‐surface trafficking of GluA1. Immunostaining with an anti‐GluA1 antibody without permeabilization revealed that Sema3A failed to increase the trafficking of GluA1 to the cell surface in the presence of the plexin A4‐IPT domain (*p* = 0.3418, *t* = 0.9671, *df* = 28) (Figure 1f). Consistent with this, Sema3A‐induced trafficking of GluA1 to the neuronal surface was attenuated by the knockdown of plexin A4 expression with lentivirus‐mediated introduction of RNA interference (RNAi) targeting plexin A4 (Figure S2), as compared to introduction of a scrambled construct, in hippocampal primary cultures (plexA4 RNAi *p* = 0.4234, *t* = 0.8078, *df* = 46; Scramble RNAi *p* = 0.0095, *t* = 2.705, *df* = 47) (Figure 1g). These results indicate that GluA1 is trafficked to the neuronal surface via IPT domain‐mediated interaction with plexin A4.

### 3.2 Knockdown of plexin A4 expression attenuates long‐term potentiation in hippocampal slices

**FIGURE 2:**
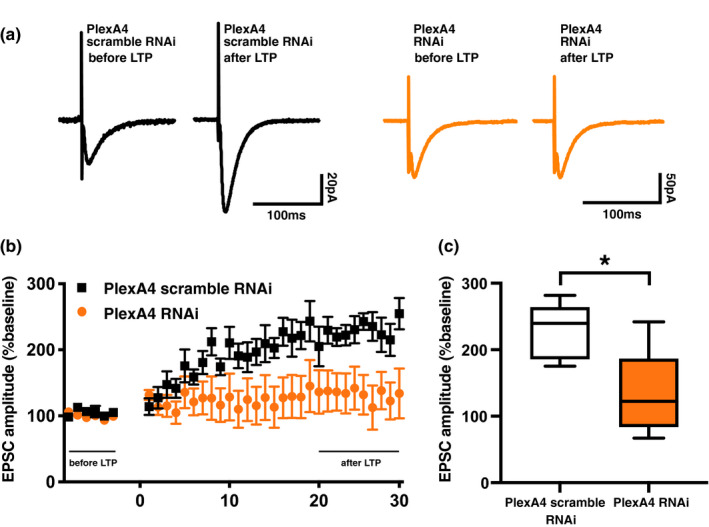
Knockdown of plexin A4 expression attenuates long‐term potentiation in hippocampal slices. Normalized responses from plexin A4 RNAi‐infected cells or scramble RNAi‐infected cells in the CA1 of the hippocampus. Long‐term potentiation (LTP) at CA1 synapses of the hippocampus was induced by pairing of 5‐Hz presynaptic stimulation with postsynaptic depolarization at 0 mV for 90 s. Recordings were maintained for at least 30 min after pairing. (a) Representative EPSC from before and after LTP induction in plexin A4 RNAi‐infected cells or scramble RNAi‐infected cells. (b) The EPSC amplitude was normalized to the average baseline amplitude before pairing. Scramble RNAi (black square) and plexin A4 RNAi (orange circle) data are shown. (c) Mean EPSC amplitude between 20 and 30 min after LTP induction. EPSC amplitude was normalized to the baseline amplitude. Scramble RNAi: *n* = 5 cells from 4 animals, Plexin A4 RNAi: *n* = 5 cells from 5 animals. *p < 0.05

As Sema3A–plexin A4 signaling regulates trafficking of GluA1 to the neuronal surface, we hypothesized that Sema3A–plexin A4 signaling is also involved in Hebbian synaptic plasticity. We expressed either an RNAi construct to knockdown plexin A4 expression or a scrambled construct in the CA1 region of the dorsal hippocampus of 4‐week‐old rats, in vivo, by lentivirus‐mediated in vivo gene transfer. One week after injection, we prepared acute hippocampal slices and induced long‐term potentiation (LTP) at CA3–CA1 pyramidal synapses using the paired protocol (0 mV, 5 Hz, and 90 s). While we found normal LTP in scramble‐RNAi expressing hippocampal slices, we detected significant attenuation of LTP in the hippocampal slices expressing the plexin A4‐knockdown RNAi construct (*p* = 0.0265, *t* = 2.713, *df* = 8; Figure 2), indicating that Sema3A–plexin A4 signaling is required for LTP induction. Thus, Sema3A–plexin A4 signaling can regulate Hebbian synaptic plasticity.

### 3.3 Fear learning induces secretion of Sema3A, which mediates memory formation

**FIGURE 3:**
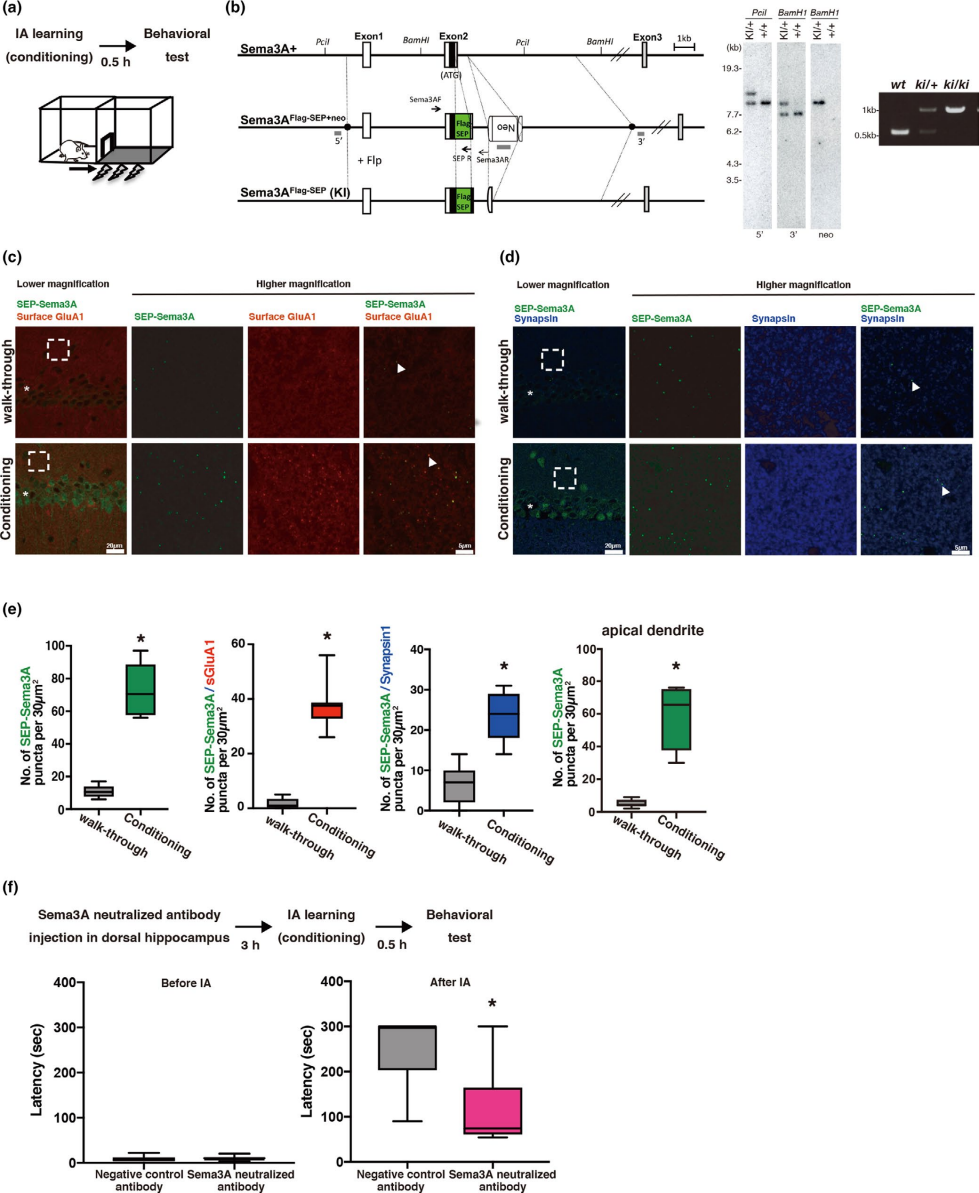
Fear learning secretes Sema3A, which mediates memory formation. (a) Experimental design and light/dark box for inhibitory avoidance (IA) task. (b) (Left) Strategy for generation of the Flag‐SEP Sema3A knock‐in mutation in mouse embryonic stem (ES) cells. Top, original wild‐type allele. Middle, targeted construct. Bottom, expected genomic structure after FLP‐mediated recombination. The position of the secretion signal coding sequence (25aa) is indicated as a black box. The 5′ and 3′ ends of the homology arm (11.92 kb) of the targeting vector are indicated as black circles. The position of the DNA probe (gray) and PCR primers (arrow) are shown. Neo, neomycin‐resistant gene expression cassette [Pgk/gb2 promoter‐driven neo‐poly(A)] flanked by two FRT elements (open semicircle). (Center) Southern blot analysis of genomic DNA in ES cells. The molecular weight marker was used to adjust the position of bands. Bands are as follows: 9.74 kb (wild type, wt) and 12.1 kb (knock‐in, KI) with 5′ probe; 7.7 kb (wt) and 9.4 kb (KI) with 3′ probe; and 9.4 kb (KI) with neo probe. Genomic DNA from original ES cells (+/+) and from recombinant ES cells (KI/+) was digested with restriction enzymes and probed. (Right) Genotype of WT, KI/+, KI/KI mice. The size of the DNA bands are as follows: wt, 550 bp; KI, 1,018 bp. (c,d) Representative photomicrograph of SEP‐Sema3A and surface GluA1(Left), or SEP‐Sema3A and synapsin (Right) in the hippocampal CA1 basal dendrite of walk‐through or IA‐conditioned Sema3A knock‐in mouse. The arrowhead indicates an example of colocalization of SEP‐Sema3A and each protein. Higher magnification of the boxed area is indicated in the right panel. The asterisk indicates the CA1 pyramidal layer. (e) Quantitative analysis of SEP‐Sema3A puncta (walk‐through: *n* = 6, conditioning: *n* = 6), colocalization of SEP‐Sema3A and surface GluA1 puncta (walk‐through: *n* = 8, conditioning: *n* = 10) or synapsin1 (walk‐through: *n* = 7, conditioning: *n* = 7) in the CA1 basal dendrite, and SEP‐Sema3A puncta (walk‐through: *n* = 6, conditioning: *n* = 6) in the CA1 apical dendrite of sema3A knock‐in mouse. “n” indicates the number of slices. (f) (Top) Experimental design for IA task using a Sema3A‐neutralizing antibody. (Bottom) Latency of entering the dark box before and after IA training in animals treated with a negative control (*n* = 6) or Sema3A‐neutralizing antibody (*n* = 7). *p < 0.05

Next, we investigated whether Sema3A mediates learning. To test this, we utilized an IA task, a hippocampus‐dependent contextual fear conditioning task (Mitsushima et al., 2011, 2013). In this task, a light box is placed next to a dark box, and rats or mice can freely move in and out of both boxes through an open door between the boxes. Soon after rats or mice enter the dark box, electric foot shocks are given. Although rats or mice usually prefer to enter a dark box, rats or mice conditioned with electric foot shocks in the dark box tend to avoid re‐entering the dark box (Figure 3a). Furthermore, we generated Flag‐SEP (super ecliptic pHluorin; a pH‐sensitive derivative of GFP)‐Sema3A knock‐in mice (Figure 3b). As SEP is pH sensitive and exhibits higher fluorescence intensity at a neutral rather than a low pH, SEP‐Sema3A shows brighter fluorescence when it is secreted. We treated animals with or without IA conditioning. Ten minutes after IA conditioning, animals were killed, their brains fixed, and hippocampal sections prepared (Figure 3c, d). We detected an increased number of SEP‐Sema3A puncta (*p* < 0.0001, *t* = 8.360, *df* = 10) that co‐localized with GluA1 puncta (*p* < 0.0001, *t* = 12.40, *df* = 16) and synapsin1 (*p* < 0.0001, *t* = 5.721, *df* = 12) compared to basal dendrite of CA1 of hippocampal sections from walk‐through animals (Figure 3e). We also detected an increased number of SEP‐Sema3A puncta in apical dendrite of CA1 of hippocampal sections (*p* < 0.0001, *t* = 6.738, *df* = 10; Figure 3e). Thus, IA learning induces secretion of Sema3A, which forms a complex with GluA1 at synapses.

Next, we examined whether secreted Sema3A mediated IA learning. We injected an anti‐Sema3A antibody, which neutralizes Sema3A function (Yamashita et al., 2015) into the CA1 region of the dorsal hippocampus of rats in vivo. Then, we conditioned the animals to the IA task. We found the latency to re‐enter the dark box was decreased in animals that received the Sema3A‐neutralizing antibody as compared to control antibody‐injected rats (*p* = 0.0309, Mann–Whitney *U* = 6) (Figure 3f). This indicated that the Sema3A‐neutralizing antibody prevents IA learning formation, and thus that IA learning‐induced Sema3A secretion mediates fear learning.

### 3.4 Sema3A–plexin A4 signaling mediates fear learning

**FIGURE 4:**
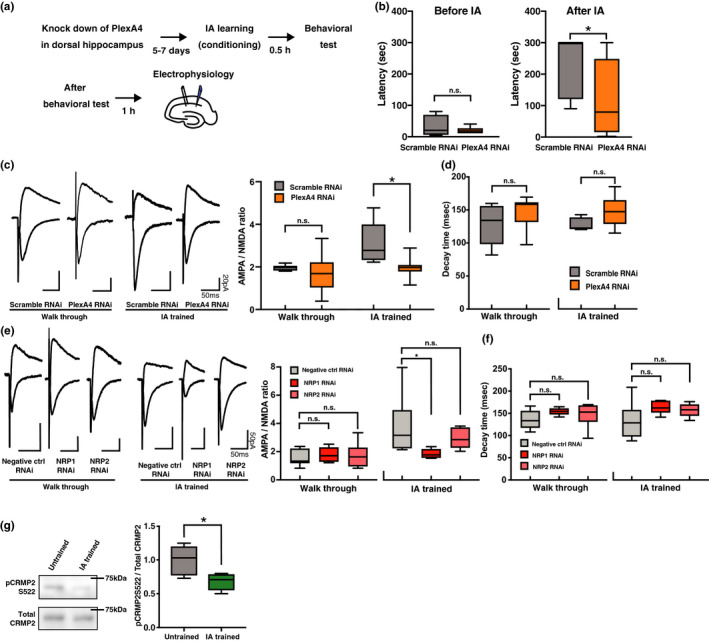
Sema3A–NRP1–plexin A4 signaling mediates fear learning. (a) Experimental design for inhibitory avoidance (IA) task using RNAi knockdown. (b) Latency of entering the dark box before and after IA training of animals expressing scramble RNAi (*n* = 7) or plexin A4 RNAi (*n* = 8). (c) (Left) Representative EPSC from walk‐through or IA‐trained rats injected with scramble RNAi or plexin A4 RNAi into the hippocampus. (Right) Average AMPA/NMDA ratio for each condition. Walk‐through scramble RNAi: *n* = 8 cells from 6 animals, walk‐through plexin A4 RNAi: *n* = 10 cells from 6 animals, IA scramble RNAi: *n* = 5 cells from 5 animals, and IA plexin A4 RNAi: *n* = 7 cells from 6 animals. (d) Average decay time of NMDAR‐mediated currents from the hippocampus of walk‐through or IA‐trained rats injected with scramble RNAi or plexin A4 RNAi. (e) (Left) Representative EPSCs from walk‐through or IA‐trained rats injected into the hippocampus with negative control RNAi, NRP1 RNAi, or NRP2 RNAi. (Right) Average AMPA/NMDA ratio for each condition. Walk‐through negative control RNAi: *n* = 7 cells from 4 animals, walk‐through NRP1 RNAi: *n* = 5 cells from 4 animals, walk‐through NRP2 RNAi: *n* = 7 cells from 4 animals, IA‐negative control RNAi: *n* = 6 cells from 4 animals, IA NRP1 RNAi: *n* = 5 cells from 5 animals, and IA NRP2 RNAi: *n* = 9 cells from 4 animals. (f) Average decay time of NMDAR‐mediated currents from walk‐through or IA‐trained rats injected into the hippocampus with negative control RNAi, NRP1 RNAi, or NRP2 RNAi. (Left) Representative immunoblots of total CRMP2 and phosphorylated CRMP2 (pCRMP2) in the post‐synaptic density fraction of hippocampal lysate obtained from untrained or IA‐trained rats. (Right) Phosphorylation level of CRMP2 at Serine 522. The ratio of pCRMP2 to CRMP2 was normalized to that in the untrained group (untrained: *n* = 6, IA trained: *n* = 6). *p < 0.05

Next, we examined whether Sema3A–NRP1–plexin A4 signaling mediates learning. To this end, we injected an RNAi construct to knockdown the expression of plexin A4 or a scramble construct, into the CA1 region of the rat dorsal hippocampus, by lentivirus‐mediated in vivo gene transfer. We then conditioned the injected animals with the IA task (Figure 4a). We found that the latency to re‐enter the dark box was significantly shorter in animals with decreased plexin A4 expression in the hippocampus than in animals that received the scramble RNAi (*p* = 0.0409, Mann–Whitney *U* = 11) (Figure 4b), indicating that plexin A4 knockdown attenuated IA learning.

We have previously reported that IA learning drives GluA1 into the CA3–CA1 pyramidal synapses of the hippocampus (Mitsushima et al., 2011, 2013; Takemoto et al., 2017). We performed whole‐cell recordings of acute hippocampal slices obtained from IA‐trained or walk‐through animals (walk‐through animals, which simply entered the experimental apparatus for IA conditioning, without any electric foot shocks), with injection of either the plexin A4‐knockdown RNAi construct or the scramble construct into the CA1 region of the hippocampus. We detected no difference in AMPA receptor‐mediated currents relative to NMDA receptor‐mediated currents (AMPA/NMDA ratio; an indicator of synaptic AMPA receptor content) between scramble construct and plexin A4 RNAi‐construct‐expressing neurons in walk‐through animals. However, the AMPA/NMDA ratio of plexin A4 RNAi‐construct‐expressing neurons exhibited a significant decrease compared to scramble RNAi‐expressing neurons in IA‐trained animals (two‐way ANOVA, main effect of RNAi *p* = 0.0148, *F*(1,26) = 6.816; main effect of IA *p* = 0.012, *F*(1,26) = 7.298; RNAi ×IA interaction *p* = 0.1059, *F*(1,26) = 2.806) (post‐hoc Sidak's multiple comparisons test; plexinA4 RNAi versus scramble RNAi in IA‐trained, *p* = 0.0209; Figure 4c). We found no difference in the kinetics of NMDA receptor‐mediated currents at CA3–CA1 hippocampal pyramidal synapses between plexin A4 RNAi‐expressing and scramble RNAi‐expressing neurons of conditioned (*p* = 0.0952, *t* = 1.842, *df* = 10) and walk‐through animals (*p* = 0.1719, *t* = 1.430, *df* = 16; Figure 4d). These results indicate that IA‐driven synaptic AMPA receptor delivery at CA3–CA1 hippocampal pyramidal synapses is mediated by plexin A4 signaling.

Thus, plexinA4 mediates IA learning by driving AMPA receptors into CA3–CA1 hippocampal synapses.

### 3.5 NRP1, but not NRP2, mediates IA learning‐induced synaptic AMPA receptor delivery

Sema3A binds to NRP1, but not NRP2 (Chen et al., 1997; Takahashi et al., 1998). Thus, we tested whether IA learning‐induced synaptic AMPA receptor delivery requires NRP1. We prepared rats injected with lentivirus expressing NRP1 RNAi (to knockdown NRP1 expression), NRP2 RNAi (to knockdown NRP2 expression), or a Negative control RNAi construct (Figure S3) into the CA1 hippocampal area, in vivo, at 4 weeks of age. One week later, the animals performed IA, after which we prepared acute hippocampal slices (30 min later). We analyzed AMPA/NMDA ratios of CA3–CA1 synapses of the dorsal hippocampus. While we detected no effect of the expression of any of the RNAi constructs on the AMPA/NMDA ratio in walk‐through animals (one‐way ANOVA, *p* = 0.7479, *F*(2,16) = 0.2959) (post‐hoc Dunett's multiple comparisons test; Negative ctrl RNAi versus NRP1 RNAi, *p* = 0.7698; Negative ctrl RNAi versus NRP2 RNAi, *p* = 0.7232), we observed a decreased AMPA/NMDA ratio in NRP1 RNAi‐, but not NRP2 RNAi‐expressing neurons, as compared to the Negative control RNAi‐expressing neurons IA‐trained animals (one‐way ANOVA, *p* = 0.0718, *F*(2,17) = 3.088) (post‐hoc Dunett's multiple comparisons test; Negative ctrl RNAi versus NRP1 RNAi, *p* = 0.043; Negative ctrl RNAi versus NRP2 RNAi, *p* = 0.3864; Figure 4e). We found no difference in the kinetics of NMDA receptor‐mediated currents among NRP1 RNAi‐, NRP2 RNAi‐, and Negative control RNAi‐expressing neurons of walk‐through animals (one‐way ANOVA, *p* = 0.2719, *F*(2,16) = 1.414) (post‐hoc Dunett's multiple comparisons test; Negative ctrl RNAi versus NRP1 RNAi, *p* = 0.2049; Negative ctrl RNAi versus NRP2 RNAi, *p* = 0.498) as well as IA‐trained animals (one‐way ANOVA, *p* = 0.1196, *F*(2,17) = 2.412) (post‐hoc Dunett's multiple comparisons test; Negative ctrl RNAi versus NRP1 RNAi, *p* = 0.1065; Negative ctrl RNAi versus NRP2 RNAi, *p* = 0.153; Figure 4f). These results indicate that Sema3A–NRP1–plexin A4 signaling mediates IA learning‐induced synaptic AMPA receptor trafficking.

### 3.6 IA learning decreases phosphorylation of CRMP2 in the hippocampus

CRMP2 is a downstream signaling molecule of the Sema3A–NRP1–plexin complex (Schmidt & Strittmatter, 2007). CRMP2 has been reported to regulate synaptic function (Jin et al., 2016). We also recently reported that CRMP2 is a primary binding site and functional target of a small compound, edonerpic maleate, which accelerates the effect of rehabilitation by facilitating experience‐dependent synaptic AMPA receptor delivery (Abe et al., 2018). Thus, we here examined whether IA learning affects the phosphorylation status of CRMP2. We prepared the PSD fraction of the dorsal hippocampus of IA‐treated or walk‐through rats. We found that IA‐treated animals exhibited decreased phosphorylation of serine 522 of CRMP2 as compared to walk‐through animals (*p* = 0.0083, *t* = 3.277, *df* = 10; Figure 4g).

## 4 DISCUSSION

The semaphorin family has attracted much attention in various biological fields, including neuroscience, immunology, and oncology (Gurrapu et al., 2018; Kolodkin & Tessier‐Lavigne, 2011; Vadasz & Toubi, 2018). In neuroscience, semaphorins have been intensively studied in terms of axon guidance and cell migration. Here, we reported that Sema3A is a crucial mediator of learning, which represents a novel role for the semaphorin family in the nervous system. IA learning, one form of a contextual fear learning task, induced Sema3A secretion, and hippocampal injection of a Sema3A‐neutralizing antibody prevented IA memory formation. Furthermore, knockdown of each component of the Sema3A receptor complex, including NRP1 and plexin A4, blocked IA learning and IA learning‐dependent synaptic AMPA receptor delivery at CA3–CA1 pyramidal synapses in the dorsal hippocampus. Our findings reveal the relationship between the molecular and cellular mechanisms that control both axon guidance and synaptogenesis, thereby providing evidence that link axon guidance molecules to neural plasticity.

In this study, we observed that IA‐induced secretion of Sema3A throughout the dendrites. This indicates that the global secretion of Sema3A could produce plastic environment which can be required for IA‐induced synaptic alterations. Consistent with this, we found that (a) knockdown of the expression of Plexin A4 attenuates the induction of LTP, (b) knockdown of the expression of PlexinA4 prevents IA learning, (c) knockdown of the expression of PlexinA4 or Neuropilin1 blocks IA‐induced trafficking of AMPARs presumably at specific synapses associated with IA learning, and (d) neuralization of Sema3A in vivo prevents IA learning. Thus, we consider that Sema3A might not directly induce synaptic potentiation driven by IA learning but rather set up the global environment required for input‐specific synaptic potentiation.

Recently, we identified a novel CRMP2‐binding small compound, edonerpic maleate, which facilitates experience‐dependent synaptic AMPA receptor delivery and accelerates motor function recovery after brain damage (Abe et al., 2018). We demonstrated that CRMP2 can be a regulator of experience‐dependent synaptic plasticity. While Sema3A signaling increases CRMP2 phosphorylation (Serine 522) in axons, CRMP2 phosphorylation at this residue was decreased in IA‐conditioned animals. Moreover, we reported that CRMP2 phosphorylation, presumably at Serine 522, was also decreased in the functional compensatory cortical area of animals that exhibited functional recovery after cortical injury due to administration of edonerpic maleate, a CRMP2‐binding compound facilitating experience‐dependent synaptic AMPA receptor delivery (Abe et al., 2018). Thus, the Sema3A–NRP1–plexin A4 complex could differentially regulate CRMP2 between axons and dendritic spines and exert diverse functions in the nervous system.

We found that Sema3A‐induced synaptic plasticity was mediated by NRP1, but not NRP2, proving the specificity of semaphorin signaling. A recent elegant study from Kolodkin's group demonstrated that Sema3F–NRP2–plexin A3 mediates downscaling of the AMPA receptor‐mediated synaptic current by global neuronal activation with bicucullin (Wang et al., 2017). Interestingly, Sema3A does not mediate this homeostatic synaptic scaling. Taken together with our finding, this suggests that Sema3A–NRP1–plexin A4 mediates Hebbian synaptic plasticity and learning/memory, while Sema3F–NRP2–plexin A3 mediates synaptic scaling, a different form of synaptic plasticity. Thus, each class III semaphorin molecule differentially and precisely regulates distinct neuronal plasticity events through its specific receptor complex. Further studies should elucidate signaling mechanisms underlying the diverse semaphorin functions in plasticity.

It is interesting that activity‐induced secretion of proteins can modify synaptic functions. Previous studies have shown that brain‐derived neurotrophic factor secretion from neurons is induced by activity, mediated by synaptotagmin 6 and complexin 1/2 (Wong et al., 2015). Additionally, global neuronal activation promotes secretion of Sema3F, which regulates synaptic downscaling (Wang et al., 2017). Here, we showed that learning induced secretion of Sema3A, and mediates learning. It will be interesting to investigate how distinct synaptic inputs induce secretion of different molecules to regulate various neuronal functions.

There are some reports that indicates Semaphorin 3A induces local protein synthesis (Cagnetta et al., 2019; Manns et al., 2012; Wu et al., 2005). Maintenance of long‐term memory requires protein synthesis to stabilize synaptic changes in the brain. It is possible that Semaphorin 3A signaling affects memory maintenance. Further experiments are needed to elucidate that Semaphorin 3A signaling affect memory formation or maintenance.

We have recently reported that Nogo, another molecule that inhibits neurite extension, restricts experience‐dependent synaptic AMPA receptor delivery (Jitsuki et al., 2016), which is opposite to the Sema3A function on synaptic AMPA receptor delivery. As Nogo and Sema3A share some signaling molecules, such as Rho (Liu & Strittmatter, 2001), how these two axonal extension inhibitory molecules differentially utilize signaling molecules to control synaptic AMPA receptor delivery should be investigated in future.

Because the CRMP2–edonerpic maleate complex mediates synaptic AMPA receptor delivery and accelerates the effect of rehabilitation after brain damage (AbStrittmattee et al., 2018), our finding further highlights Sema3A–CRMP signaling cascades as a target for potential pharmacological intervention.

## CONFLICTS OF INTEREST

The authors declare that no competing interests exist.

## AUTHOR CONTRIBUTIONS

T.T. designed the whole project and wrote the manuscript. Y.G. designed the experiments regarding the role of secretion of Sema3A. A.J‐T. and S.J. designed the experiments, conducted experiments (in vitro immunostaining, electrophysiological experiments, and behavioral experiments), and wrote the manuscript. N.Y., M.K., M.A., and K.S. produced SEP‐Sema3A knock‐in mice. A.S. produced viral constructs and preparation. All authors read and approved submission of the final version of the manuscript.

### PEER REVIEW

The peer review history for this article is available at https://publons.com/publon/10.1111/ejn.15210.

## Supporting information

Fig S1‐S3

## Data Availability

The datasets generated and analyzed during the current study are available from the corresponding author on reasonable request.
