# Supplementary material for: Activity‐induced secretion of semaphorin 3A mediates learning
Source: Eur J Neurosci. 2021 Apr 5;53(10):3279–93. doi: 10.1111/ejn.15210 (PMC8252788; doi:10.1111/ejn.15210)
Supplement: Supplementary file 1 — Fig S1‐S3 [file EJN-53-3279-s001.docx]

**Supplementary Results**


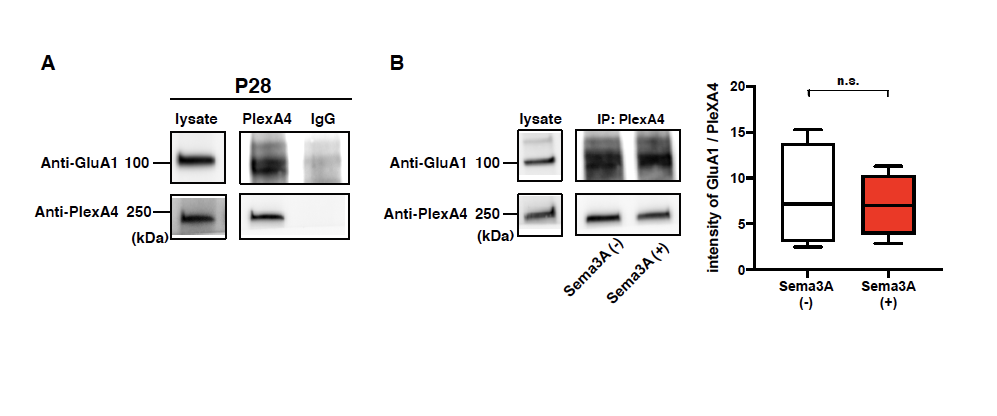


Supplementary Figure1. Immunoprecipitation of GluA1 and plexin A4.

(A) Immunoprecipitation of GluA1 and plexin A4 at postnatal day (P) 28 rat hippocampal lysates. GluA1 and plexin A4 were co-immunoprecipitated from rat hippocampal lysates at P28.

(B)(left) Immunoprecipitation of GluA1 and plexin A4 in the presence or absence of sema3A from DIV21 primary hippocampal neurons. (right) Quantification of GluA1-plexin A4 complex. Plexin A4 was the reference amount for the quantitative analysis. Data were analyzed by unpaired t-test.


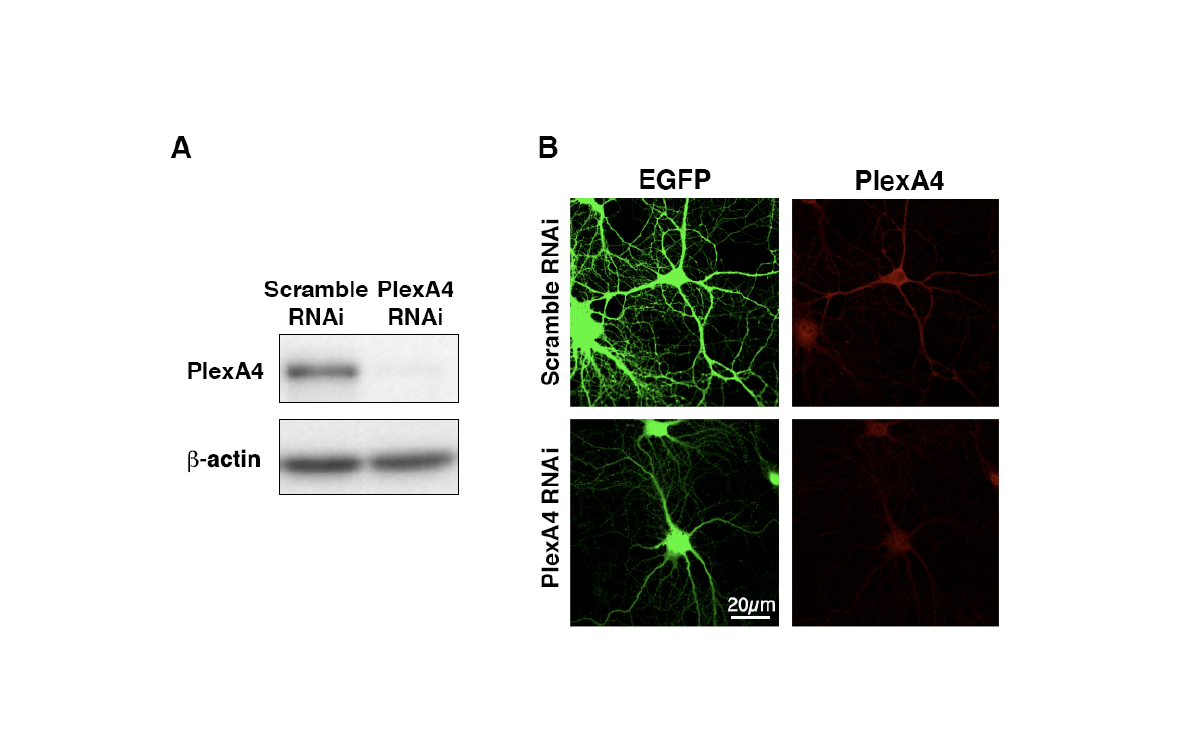


Supplementary Figure2. Knock down of PlexA4 in cultured neuron.

(A) Immunoblot of PlexA4 and β−actin obtained from lysate of PlexA4 RNAi or scrambled RNA infected cultured neurons at DIV8. The PlexA4 RNAi or scrambled RNA were expressed by lentivirus-mediated gene transfer at DIV1.

(B) Representative images of PlexA4 RNAi or scrambled RNA infected neurons. The PlexA4 RNAi or scrambled RNA was co-expressed with EGFP. The neurons were stained with PlexA4 antibody.


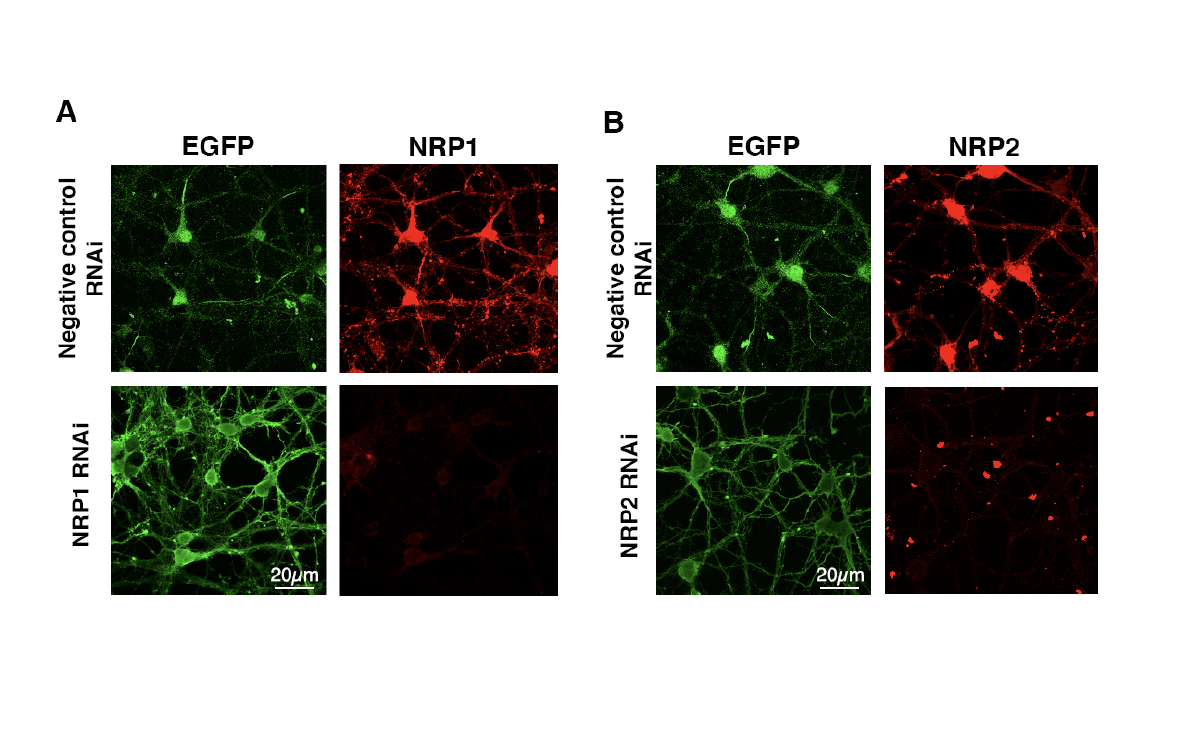


Supplementary Figure3. Knock down of NRP1 and NRP2 in cultured neuron.

(A) Representative images of NRP1 RNAi or Negative control RNAi infected cultured neurons. The NRP1 RNAi or Negative control RNAi was co-expressed with EGFP. The neurons were stained with NRP1 antibody.

(B) Representative images of NRP2 RNAi or Negative control RNAi infected cultured neurons. The NRP2 RNAi or Negative control RNAi was co-expressed with EGFP. The neurons were stained with NRP2 antibody.
